# CT changes of severe coronavirus disease 2019 based on prognosis

**DOI:** 10.1038/s41598-020-78965-0

**Published:** 2020-12-14

**Authors:** Bin Liang, Lingli Xie, Fan Yang, Joyman Makamure, Lijie Zhang, Ran Pang, Peng Du, Wenhui Fan, Chuansheng Zheng

**Affiliations:** 1 Department of Radiology, Hubei Key Laboratory of Molecular Imaging, Union Hospital, Tongji Medical College, Huazhong University of Science and Technology, Wuhan, China; 2 Department of Respiratory Medicine, General Hospital of the Yangtze River Shipping, Wuhan, China; 3 Department of Infectious Diseases, Union Hospital, Tongji Medical College, Huazhong University of Science and Technology, Wuhan, China; 4 Department of Radiology, General Hospital of the Yangtze River Shipping, Wuhan, China

**Keywords:** Infectious diseases, Medical research

## Abstract

This study aimed to determine the characteristics of CT changes in patients with severe coronavirus disease 2019 (COVID-19) based on prognosis. Serial CT scans in 47 patients with severe COVID-19 were reviewed. The patterns, distribution and CT score of lung abnormalities were assessed. Scans were classified according to duration in weeks after onset of symptoms. These CT abnormalities were compared between discharged and dead patients. Twenty-six patients were discharged, whereas 21 passed away. Discharged patients were characterized by a rapid rise in CT score in the first 2 weeks followed by a slow decline, presence of reticular and mixed patterns from the second week, and prevalence of subpleural distribution of opacities in all weeks. In contrast, dead patients were characterized by a progressive rise in CT score, persistence of ground-glass opacity and consolidation patterns in all weeks, and prevalence of diffuse distribution from the second week. CT scores of death group were significantly higher than those of discharge group (*P* < 0.05). The CT changes differed between the discharged and dead patients. An understanding of these differences can be of clinical significance in the assessment of the prognosis of severe COVID-19 patients.

## Introduction

An outbreak of coronavirus disease 2019 (COVID-19) was first reported in Wuhan, China in December 2019^1^, and the pandemic has accelerated recently, with a considerable number of cases now confirmed in multiple countries and territories. This disease is caused by the severe acute respiratory syndrome coronavirus 2 (SARS-CoV-2), and has been regarded as a life-threatening respiratory infection^2^. By August 20th, 2020, 68,139 cases had been confirmed, with 4512 deaths in Hubei Province, China^3^. Although COVID-19 may present with mild, moderate or severe illness, a minority of patients with severe illness may deteriorate rapidly and even die, and these patients will require accurate assessment and aggressive treatment^4^.

CT is currently the preferred imaging modality employed for patients with COVID-19 in China. Besides diagnosing the novel coronavirus pneumonia and planning its treatment^5–8^, CT has played an important role in evaluating prognosis, particularly in severe COVID-19 patients. A recently published study compared CT findings in critically ill patients with COVID-19 who survived and died and found that patients with diffuse lung involvement were more likely to die of COVID-19^9^. Another recent publication investigated risk factors for mortality and found that CT severity score is a reliable predictor of mortality in nonelderly previously healthy individuals with COVID-19^10^. However, the previous two studies only included one chest CT scan for each patient, one of which indicated that the CT scan was performed within 24 h when the patient met the clinical severity criteria^9^. Knowledge of dynamic changes in CT findings may be of more value to guide management and assess prognosis. Therefore, the aim of this study was to determine the characteristics of CT changes of severe COVID-19 between recovered and dead patients.

## Materials and methods

### Patients

The Medical Ethics Committee of Wuhan Union Hospital, Tongji Medical College, Huazhong University of Science and Technology and General Hospital of the Yangtze River Shipping (Wuhan, China) approved this study and waived informed consent. The study was carried out in accordance with the principles embodied in the Declaration of Helsinki. A retrospective analysis was conducted of all patients who had been hospitalized for COVID-19 infection at the Wuhan Union Hospital and the General Hospital of the Yangtze River Shipping from December 25 2020 to March 12 2020. The criteria for patient selection included (1) patients aged > 18 years, (2) patients whose pharyngeal swab specimen tested positive for COVID-19 by real-time RT-PCR, (3) patients who presented with severe illness, (4) patients who had been discharged from hospital or had died of the disease, and (5) patients who underwent at least two serial chest CT scans. Patients were excluded if they had been transferred to other hospitals, had mild or moderate illness, or had incomplete CT or clinical data.

According to the WHO guidance^4^, severe COVID-19 infection includes severe pneumonia and the relevant clinical syndromes, including acute respiratory distress syndrome (ARDS), sepsis and septic shock. The severe pneumonia is defined as fever or suspected respiratory infection, plus one of respiratory rate > 30 breaths/min, severe respiratory distress, or SpO_2_ < 90% on room air. The definition of the other 3 clinical syndromes, which developed based on the pneumonia, are also referenced in the WHO guidance^4^.

The criteria for patient discharge include being afebrile for greater than 3 days, significant improvement in respiratory symptoms and radiological abnormalities, and two consecutive pharyngeal swab specimens testing negative for COVID-19 at least 24 h apart^11^.

### CT imaging

Chest CT examinations were performed using multidetector CT scanners (Somatom Perspective; Somatom Spirit; Somatom Definition AS+, Siemens Healthineers, Germany; Aquilion one, Toshiba, Japan). The patients received non-contrast enhanced CT scanning on breath-hold in the supine position and the scan ranged from the level of the thoracic inlet to the costophrenic angles. The CT parameters were as follows: tube voltage, 120 kV; tube current, regulated by an automatic exposure control system. Images were reconstructed at 1.5-mm slice thickness and interval, and then transmitted to picture archiving and communication systems (PACS) for interpretation or additional post-processing^5^.

### Image evaluation

The serial thin-section CT images of the patients during hospitalization were reviewed in consensus by two radiologists (B.L. and F.Y., with 26 and 12 years of experience in diagnostic radiology, respectively), who were blinded to the patients’ demographic, clinical and laboratory data.

Each lobe of the lung was reviewed for possible abnormal findings in accordance with the glossary of terms for thoracic imaging recommended by Fleischner Society^12^. The predominant patterns of abnormality on CT scans were categorized as ground-glass pattern, consolidation pattern, reticular pattern and mixed pattern^13^. Ground-glass opacity pattern appeared as ground-glass opacities alone or with superimposed interlobular and intralobular septal thickening and irregular linear opacities. Consolidation pattern appeared as consolidation alone or predominant consolidation without architectural distortion. Reticular pattern consisted of either coarse linear or curvilinear opacities or fine subpleural reticulation without substantial ground-glass opacities. Mixed pattern appeared as a combination of consolidation, ground-glass opacities, and reticular opacities in the presence of architectural distortion^13^. Pleural thickening, pleural effusion, mediastinal lymphadenopathy, pneumothorax, pneumomediastinum and other possible findings were also recorded.

The distribution of opacities was evaluated according to the previous method^13^ with minor modifications. Opacities were noted as being subpleural (abutting the pleural surface, including interlobar pleura), random (without predilection for subpleural or central regions), or diffuse (continuous involvement without respect to lung segments).

The extent of lung lesion was also quantified according to a CT scoring system^14^. Each lobe of the lung was visually scored on a scale of 0–5, depending on the percentage of each lobe involved: 0, no involvement; 1, < 5%; 2, 5–25%; 3, 26–49%; 4, 50–75%; 5, > 75%. The sum of scores of the 5 lobes provided total lung involvement, with a range of 0–25.

### Collection of clinical data

Data for demographic, clinical and laboratory parameters on admission, including age, sex, symptoms, comorbidities, clinical syndromes, lymphocyte count, C-reactive protein (CRP), lactate dehydrogenase (LDH), alanine aminotransferase (ALT), and pulse oxygen saturation (SpO_2_) levels were collected by one of three clinicians (L.L.X., R.P., and P.D.) for evaluating severity of illness. Management of the patients, including supportive treatment, antiviral therapy, respiratory support, organ function support, blood purification, immunomodulating therapy, etc., was conducted according to the diagnosis and treatment guidelines for COVID-19 established by National Health Commission of the People’s Republic of China^11^. Patient prognosis, either discharge or death, was also documented.

### Statistical analysis

Statistical analyses were performed using IBM SPSS Statistics (version 22; SPSS, Chicago, Ill). The data were expressed as Mean ± SD and median and range unless otherwise stated. Differences in CT parameters between discharged and dead patients were tested using Chi-square test and Mann–Whitney *U* test. Two-sided *P* < 0.05 was considered statistically significant.

## Results

### Patient characteristics

A total of 498 patients had been hospitalized for COVID-19 by the end of March 20 2020. 451 patients were excluded due to transfer to other hospitals (204 patients), mild or moderate illness (222 patients), or incomplete data (25 patients). Consequently, 47 patients were included in this study.

Of the included 47 patients, 26 patients were discharged after treatment, whereas 21 patients died from the severe illness. Time from onset of symptoms to discharge or death was 27.4 ± 8.3 (27, 12–50) days or 17.9 ± 7.3 (16, 8–33) days, respectively. Initial symptoms included fever, fatigue, dry cough, expectoration, pharyngalgia, dyspnea, anorexia, myalgia, diarrhea, nausea and vomiting. Table 1 summarizes the baseline characteristics and clinical syndromes of patients with severe COVID-19 based on prognosis. The dead patients showed significant increases in age, comorbidities (cerebrovascular disease, diabetes mellitus and chronic kidney disease) and clinical syndromes (sepsis and septic shock) compared with the discharged patients (*P* < 0.05). In addition, the dead patients showed significant decreases in lymphocyte count (*P* < 0.001), CRP (*P* < 0.001), ALT (*P* < 0.001) and SpO_2_ level (*P* < 0.001) compared with the discharged patients at baseline.

**Table 1:** Baseline characteristics and clinical syndromes of patients with severe COVID-19.

Parameters	Discharge patients (n = 26)	Dead patients (n = 21)	*P* value
Age	61.7 ± 14.8 (60, 24 − 90)	77 ± 12.5 (78, 56 − 99)	.001
**Sex (man/woman)**			.770
Female	11 (42.3%)	8 (38.1%)	
Male	15 (57.7%)	13 (61.9%)	
**Comorbidities**	19 (73.1%)	20 (95.2%)	.044
Hypertension	15 (57.7%)	12 (57.1%)	.970
Cardiovascular disease	7 (26.9%)	11 (52.4%)	.074
Cerebrovascular disease	0	4 (19%)	.020
Diabetes mellitus	2 (7.7%)	9 (42.9%)	.005
COPD	1 (3.8%)	2 (9.5%)	.429
Chronic kidney disease	1 (3.8%)	6 (28.6%)	.018
Chronic hepatic disease	7 (26.9%)	6 (28.6%)	.900
Others	4 (15.4%)	3 (14.3%)	.916
Lymphocyte count (× 10^9^/L)	1.1 ± 1.3 (0.7, 0.4 − 7.5)	0.9 ± 0.4 (1, 0.2 − 2)	< .001
CRP level (mg/L)	36.1 ± 41.4 (27.5, 4.7 − 187.8)	30.8 ± 15.5 (31.3, 4.2 − 83.6)	< .001
LDH level (U/L)	330 ± 129.5 (301, 172 − 641)	312.2 ± 98.6 (302, 134 − 550)	.323
ALT level (U/L)	33.4 ± 25.3 (26, 8 − 116)	24.6 ± 16.4 (21, 8 − 70)	< .001
SpO_2_ level (%)	91.6 ± 7.3 (94, 64 − 99)	88.1 ± 8.2 (89, 69 − 98)	< .001
**Clinical syndromes**			
Severe pneumonia	26	21	−
ARDS	21 (80.8%)	20 (95.2%)	0.139
Sepsis	11 (42.3%)	19 (90.5%)	.001
Septic shock	3 (11.5%)	20 (95.2%)	< .001

Except where otherwise indicated, data are mean ± SD (median and range) of age or number (%) of patients.

*COPD* chronic obstructive pulmonary disease, *CRP* C-reactive protein, *LDH* lactate dehydrogenase, *ALT* alanine aminotransferase, *SpO*_*2*_ pulse oxygen saturation, *ARDS* acute respiratory distress syndrome.

### Changes of CT abnormalities

The indications for serial scans included initial diagnosis, clinical deterioration and requirement of a change in treatment. The mean number of CT scans was 3.3 ± 1.2 (3, 2–6) per patient. The mean time from onset of symptoms to the first CT scan was 4.9 ± 3 (5, 0–13) days, and the mean time from the last CT scan to discharge or death was 3.3 ± 2.3 (3, 0–10) and 5.6 ± 4 (5, 0–18) days, respectively.

**Figure 1:**
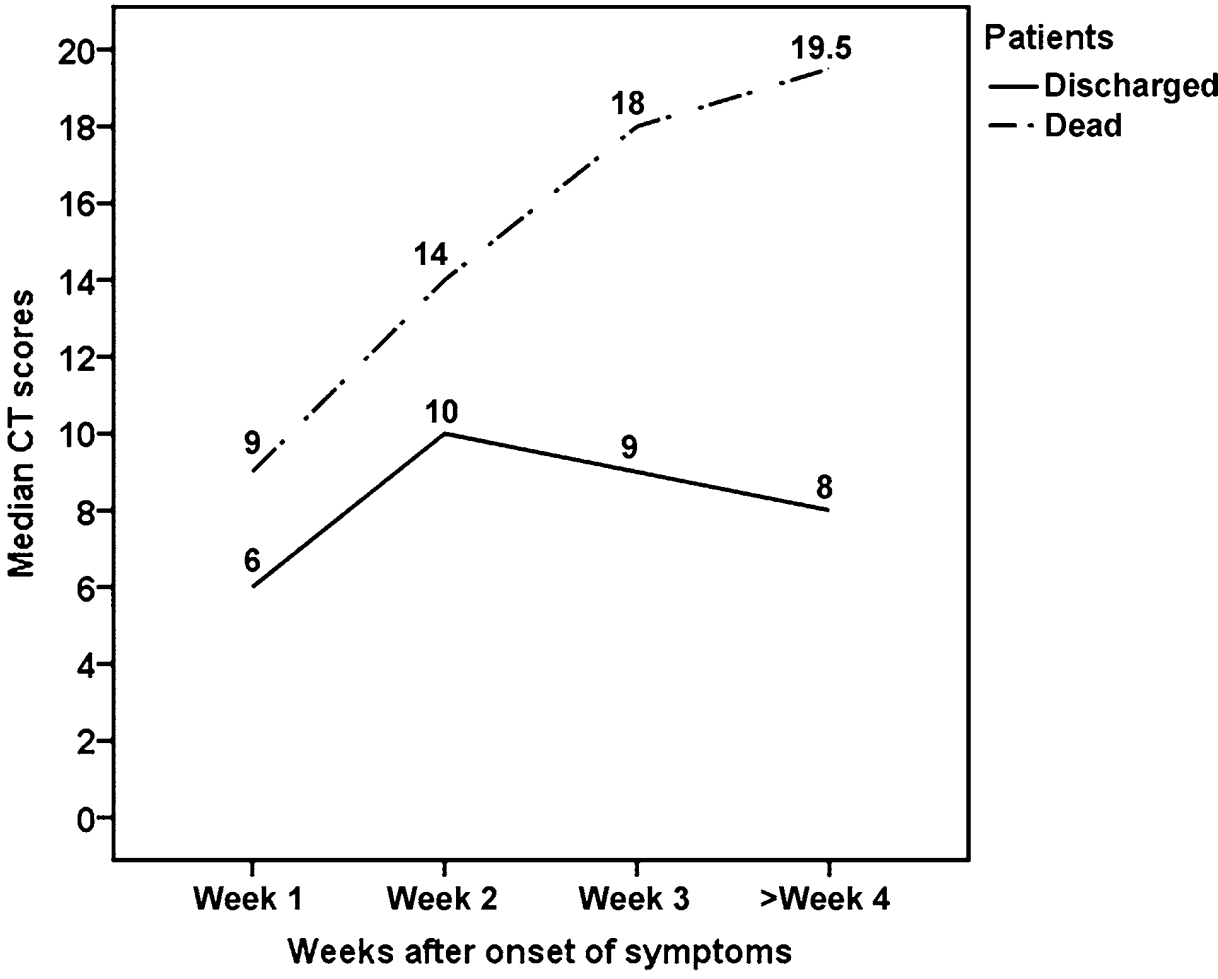
Line graph shows median CT scores in discharged and dead patients on CT scans at different time points after onset of symptoms.

There were apparent changes of CT abnormalities in patients with severe COVID-19 during the hospitalization. In the 26 discharged patients, the median CT score was markedly increased from 6 (rang, 0–11) in the first week to 10 (range, 5–21) in the second week (*P* < 0.001), and then it dropped slowly to 9 (range, 5–17) and 8 (range, 3–15) in the third (*P* = 0.586) and the fourth week or longer (*P* = 0.068), respectively (Fig. 1).

**Figure 2:**
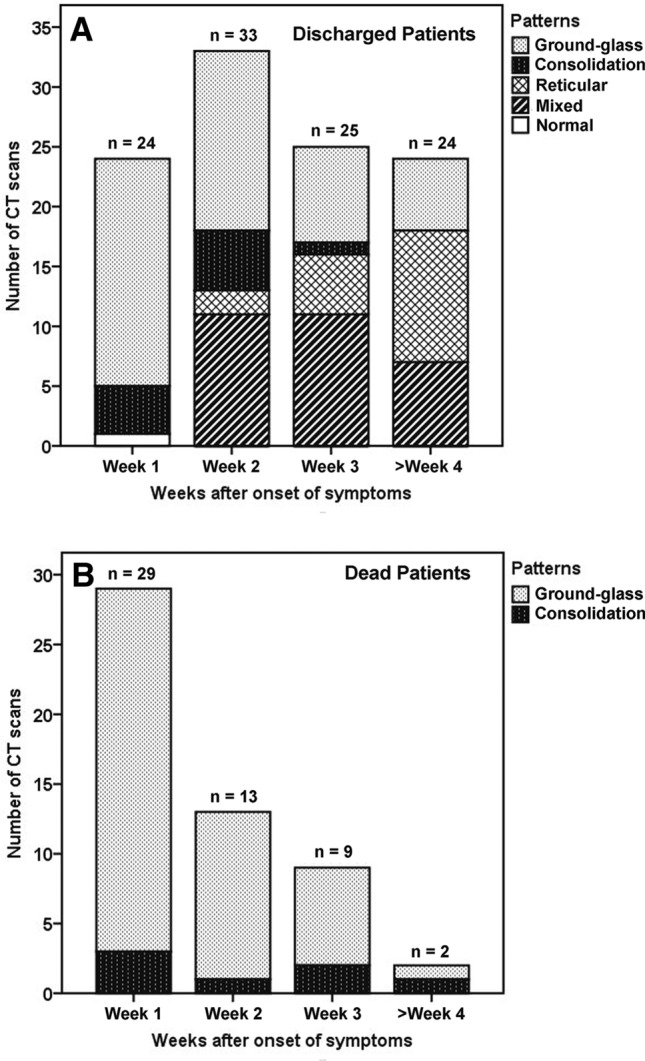
Stacked-bar graphs show distribution of various patterns of lung abnormalities in discharged **(A)** and dead patients **(B)** on CT scans at different time points after onset of symptoms.

**Figure 3:**
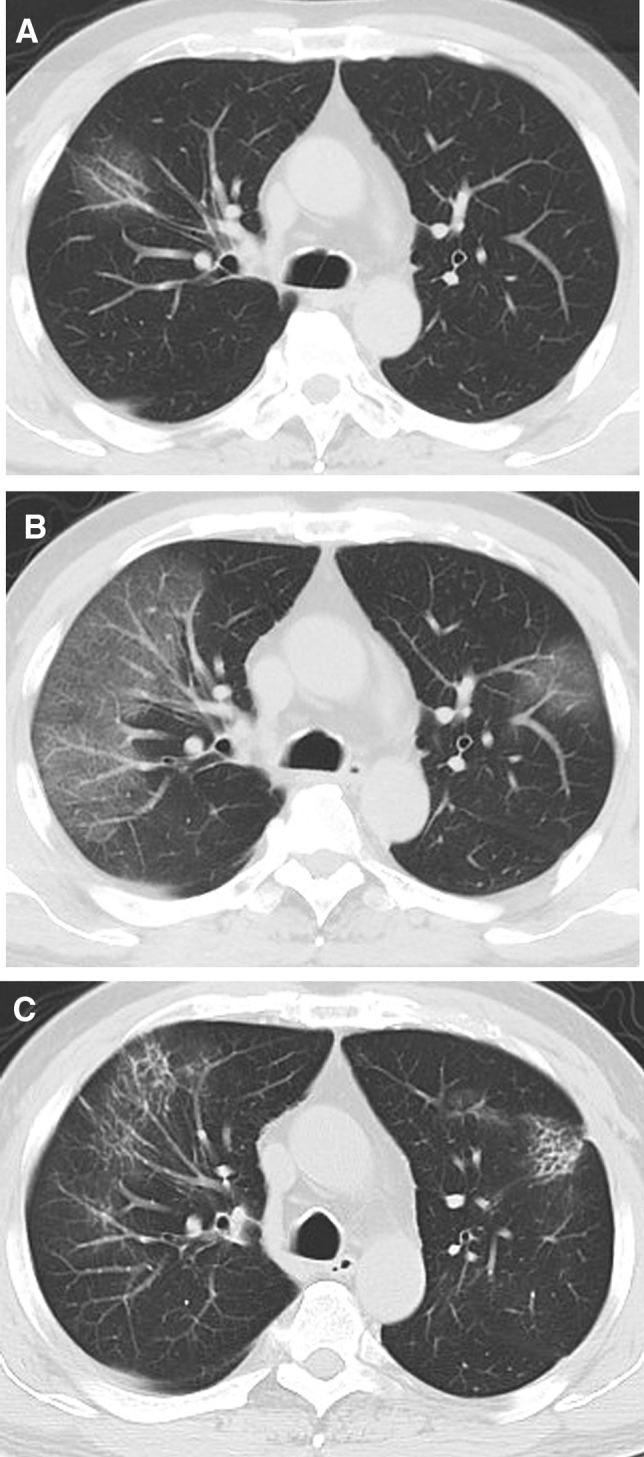
CT findings of a representative COVID-19 patient in discharge group who presented with fever and fatigue, severe pneumonia, and improved in symptoms corresponding to the CT scans. **(A)** Scan obtained on illness day 3 shows focal ground-glass opacities in right upper lobe, with a random distribution. **(B)** Scan obtained on illness day 8 shows that the ground-glass opacities were increased. **(C)** Scan obtained on illness day 18 shows development of a residual reticular pattern.

**Figure 4:**
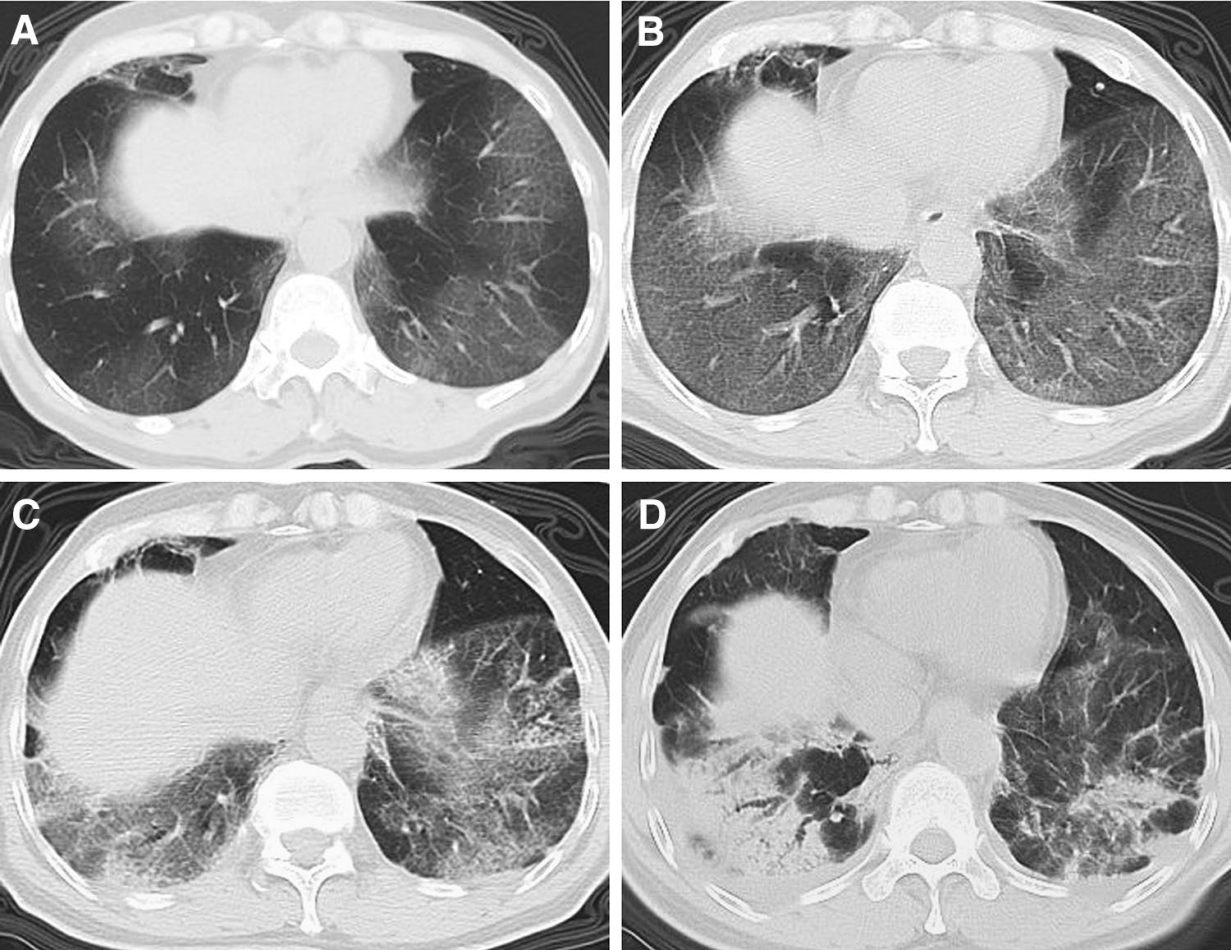
CT findings of a representative COVID-19 patient in discharge group who presented with fever and mild dyspnea, severe pneumonia, and gradually improved in symptoms corresponding to the CT scans. **(A)** Scan obtained on illness day 4 shows multifocal ground-glass opacities in double lower lobes, with a subpleural distribution. **(B)** Scan obtained on illness day 7 shows that the ground-glass opacities were increased, with superimposed interlobular and intralobular septal thickening and with diffuse distribution. **(C)** Scan obtained on illness day 12 shows that the ground-glass opacities were decreased, with superimposed irregular opacities. **(D)** Scan obtained on illness day 23 shows development of a mixed pattern.

The predominant abnormalities were ground-glass opacity and opacification within the first week, followed by coexistence of 4 patterns during the second week, after which the pattern appeared as ground-glass, reticular or mixed patterns (Fig. 2A). The frequency of the ground-glass pattern (Figs. 3 and 4) was highest in the first week (79.2%, 19/24) and maintained a high proportion in the second week (45.5%, 15/33), after which it decreased. Superimposed interlobular and intralobular septal thickening (Fig. 4B) were frequently observed in the first 2 weeks and superimposed irregular linear opacities (Fig. 4C) became more common thereafter. Consolidation pattern was not common, with a frequency of 16.7% (4/24), 15.2% (5/33) and 4% (1/25) in the first, second and third week, respectively, although consolidation was frequently noted in combination with other abnormalities. Reticular pattern (Fig. 3C) was found from the second week (6.1%, 2/33) and became more common in the third (20%, 5/25) week and fourth week or longer (45.8%, 11/24). Mixed pattern (Fig. 4D) was noted from the second week and maintain high proportions in the second (33.3%, 11/33) week and the third week (44%, 11/25), after which it decreased (29.2%, 7/24). In terms of the longitudinal changes of abnormalities, the initial CT scans demonstrated predominant ground-glass opacities, consolidation, mixed pattern and normal findings in 20, 4, 1 and 1 patients, respectively. Of the 20 patients with ground-glass opacities on the initial scans, 10 developed a reticular pattern, 8 developed a mixed pattern, and 2 decreased in extent before discharge. Of the 4 patients with consolidation on the initial scans, 2 developed a ground-glass opacity pattern, 1 developed a reticular pattern, and 1 developed a mixed pattern. The patient with mixed pattern on the initial scan revolved completely, and the patient with normal findings developed a reticular pattern. Of note, the reticular and mixed pattern generally occurred in the background of the original ground-glass opacities or consolidation and may persist until discharge (Figs. 3 and 4).

**Figure 5:**
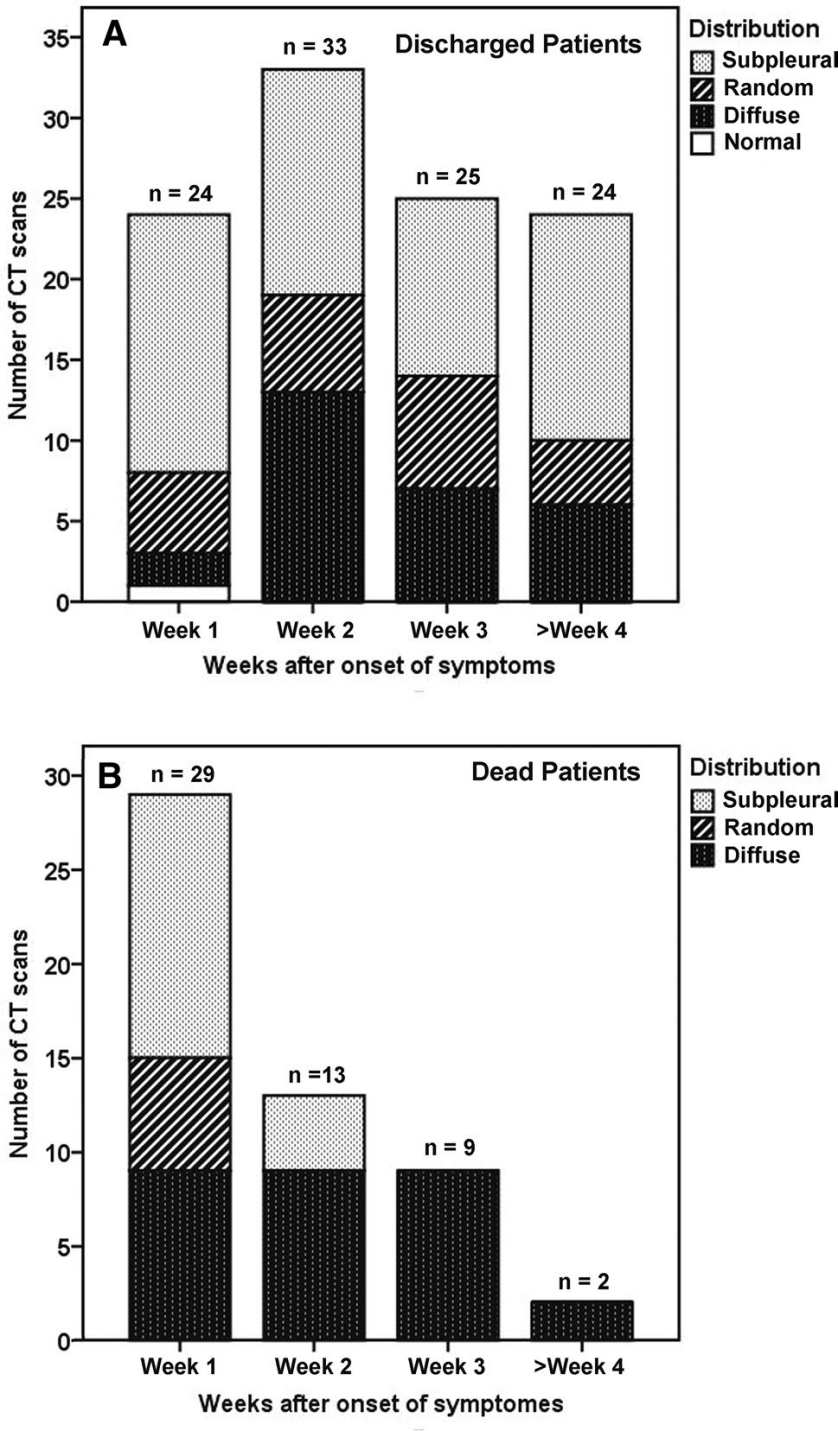
Stacked-bar graphs show distribution of various distribution of opacities in discharged **(A)** and dead patients **(B)** on CT scans at different time points after onset of symptoms.

The distribution of opacities also varied with time (Fig. 5A). Subpleural distribution (Fig. 4A) was predominant in all the weeks, with proportions of 42.4–66.7%. Random distribution (Fig. 3A) was noted with a low frequency in all the weeks. Diffuse distribution (Fig. 4B) was occasionally noted in the first week (8.3%, 2/24) and become more common in the second week (39.4%, 13/33), after which it decreased. Thirteen patients presented with pleural thickening adjacent to the lung abnormalities. Seven patients developed mild to mediate pleural effusion, one of whom developed concomitant pericardial effusion. Two patients developed subsegmental atelectasis, which appeared as parenchymal bands and was reversed with inflammation resolution. No pneumothorax, pneumomediastinum or mediastinal lymphadenopathy was noted.

**Figure 6:**
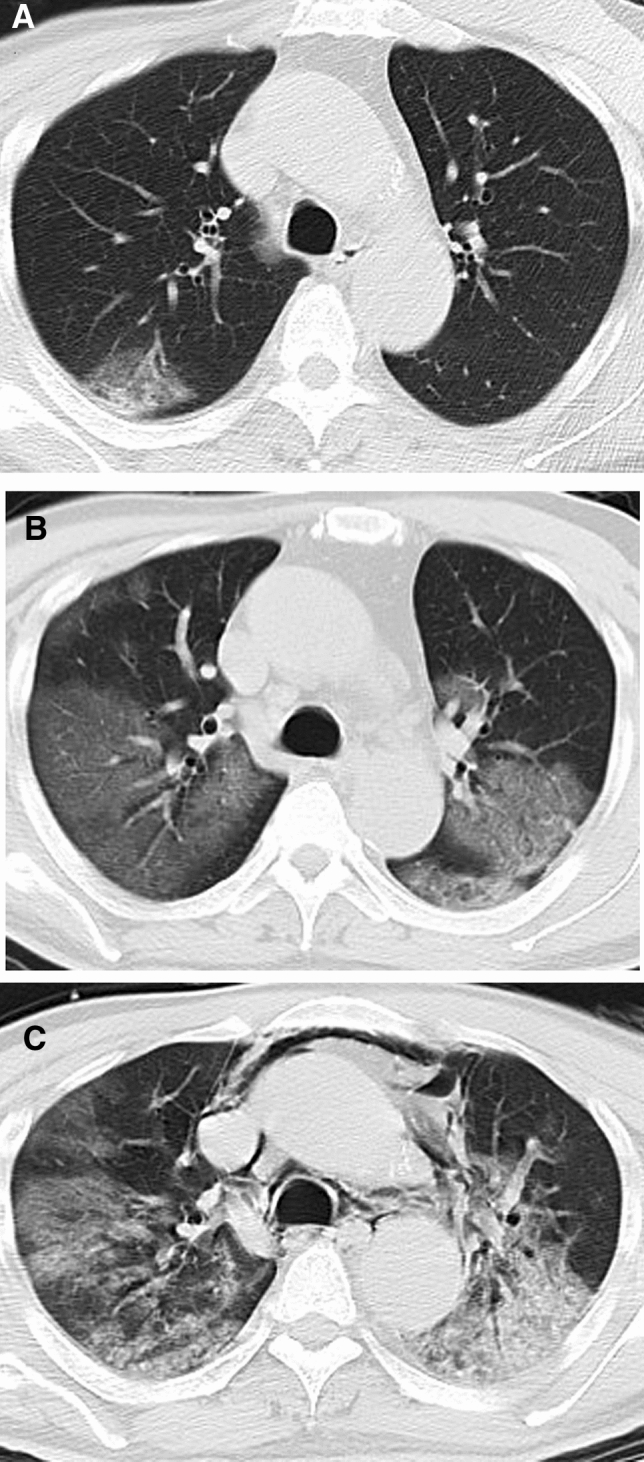
CT findings of a representative COVID-19 patient in death group who presented with fever and fatigue, severe pneumonia, and ARDS and sepsis corresponding to the CT scans. **(A)** Scan obtained on illness day 6 shows focal ground-glass opacities in right upper lobe, with a subpleural distribution. **(B)** Scan obtained on illness day 14 shows that the extent of ground-glass opacities obviously increased, with involvement of multilobes. **(C)** Scan obtained on illness day 21 shows the transformation from ground-glass opacities to consolidation, with a pneumomediastinum.

In contrast, the 21 dead patients showed different CT features (Fig. 6). The median CT score was progressively increased from 9 (range, 1–19) in the first week to 19.5 (range, 19 − 20) in the fourth week (Fig. 1). The differences in CT score between the first, the second, and the third and fourth weeks were significant (*P* < 0.05). The predominant patterns of abnormality only included ground-glass opacity and consolidation. Ground-glass opacity pattern was more common than consolidation pattern during the first 3 weeks (*P* < 0.05), and thereafter the two patterns were found in equal proportions (Fig. 2B). Opacities were predominantly distributed in the subpleural regions (48.3%, 14/29) during the first week and became more diffuse (69.2%, 9/13) in the second week, after which opacities were only found displaying a diffuse pattern (Fig. 5B). Pleural thickening and pleural effusion were found in 4 and 7 patients, respectively. Pneumomediastinum was noted in 1 patient late in the course (Fig. 6C).

### Comparison of CT changes in discharged and dead patients

Given the length of hospital stay, the distribution of time to CT scan and the CT findings, the CT changes was compared in discharged and dead patients on the bases on scans of three time periods: within the first week, within the second week, and from the third week onwards. Table 2 summarizes the temporal change in CT score, abnormality pattern and opacity distribution between the two groups of patients. CT scores of the death group were significantly higher than those of the discharge group within the first week (9 vs. 6, *P* = 0.014), the second week (14 vs. 10, *P* = 0.042) and from the third week (19 vs. 9, *P* < 0.001). The CT score was progressively increased in death group, whereas it decreased after the second week in discharged group (Fig. 1). There were significant differences in abnormality pattern between two groups within the second week (*P* = 0.029) and from the third week (*P* < 0.001). Mixed and reticular patterns were noted in charged patients during the second week and became more common thereafter, whereas they were never found in dead patients (Fig. 2). Significant differences were also noted in opacity distribution between the two groups from the third week (*P* < 0.001). Subpleural and random distribution were prevalent in the first and from the third week in discharged patients, whereas diffuse distribution achieved a dominant proportion from the second week onwards in death group (Fig. 5).

**Table 2:** Comparison of CT findings in discharge and death groups based on CT scans.

Parameters	Within the first week				Within the second week				From the third week onwards		
	Discharge (scans = 24)	Death (scans = 29)	*P* value		Discharge (scans = 33)	Death (scans = 13)	*P* value		Discharge (scans = 49)	Death (scans = 11)	*P* value
CT score	6, 0–11 (5.7 ± 3)	9, 1–19 (9.5 ± 5.3)	.014		10, 5–21 (11.2 ± 4)	14, 6–23 (14.4 ± 5.1)	.042		9, 3–17 (9.2 ± 3.4)	19, 16–22 (18.9 ± 2.0)	< .001
**CT pattern**			.460				.029				< .001
Ground-glass	19	26			15	12			14	8	
Consolidation	4	3			5	1			1	3	
Reticular pattern	0	0			2	0			16	0	
Mixed pattern	0	0			11	0			18	0	
**CT distribution**			.133				.110				< .001
Subpleural	16	14			14	4			25	0	
Random	5	6			6	0			11	0	
Diffuse	2	9			13	9			13	11	

Except where otherwise indicated, data are median and range (mean ± SD) of CT score or number of CT scans. One patient showed no any abnormalities on CT scan within the first week.

## Discussion

Management of patients with severe COVID-19 currently represents a challenge. Since the natural history of COVID-19 is not clearly understood, identification and assessment of severely ill patients is considerably based on the combination of clinical, laboratory and imaging findings^11^. Despite a number of publications regarding CT findings of severe COVID-19 have been recently reported^9,10,15–19^, the comparison of CT dynamic changes in patients with different prognosis is still lacking. In this study, we included series CT scans of patients with severe COVID-19 and classified the CT scans according to duration in weeks after onset of symptoms. Our results demonstrated that the CT changes differed between the discharged and dead patients.

Our study found that the discharged patients were characterized by a rapid rise in CT score in the first 2 weeks followed by slow decline in it, presence of reticular and mixed patterns from the second week, and prevalence of subpleural and random distribution of opacities in the first and from the third week. These findings provide a supplement to those observed in mild and moderate illness. Pan et al^6^ investigated CT changes of non-severe COVID-19 from diagnosis until recovery of disease and revealed four stages of CT characteristics. Abnormalities included ground-glass opacities and superimposed crazy-paving pattern, with mean CT scores of 2 and 6 on stage I (0–4 days) and II (5–8 days), respectively, and became more consolidative on stage III (9–13 days), with a mean peak CT score of 7, after which the consolidation resolved gradually, with a mean CT score of 6 before discharge (stage IV, ≥ 14 days). In this study, the discharged patients also showed a rapid increase in CT score and abnormality patterns of ground-glass opacities and consolidation within the first 2 weeks. However, the CT score seemed higher, with a median peak CT score of 10 in the second week, which then decreased more slowly, with median CT scores of 9 and 8 in the third and fourth week, respectively. In addition, reticular and mixed patterns were noted in the second week and became more prevalent thereafter. Diffuse distribution was also more common, particularly in the second week. Extrapulmonary abnormalities including pleural thickening, pleural effusion, subsegmental atelectasis and pneumomediastinum, were also found in this study. The discrepancies between our findings and previous findings are most likely due to differences in severity of illness.

More importantly, our study revealed the CT changes in the dead patients. They were characterized by a progressive rise in CT score, persistence of ground-glass opacities and consolidation patterns in all weeks, and prevalence of diffuse opacity distribution from the second week. By comparison of CT findings in discharged and dead patients, we found several crucial differences between the two groups, which may be helpful in the assessment of prognosis. Firstly, the extent of parenchymal abnormalities was significantly higher in the death compared with discharge group in all weeks, which suggests that the dead patients presented with a more intense inflammation storm in lungs. Accordingly, more clinical syndromes, such as sepsis and septic shock, developed and the patients deteriorate rapidly. Secondly, neither the mixed nor reticular pattern was noted in the death group, particularly from the second week. As noted in previous SARS patients, the mixed and reticular patterns probably represent improvements of pneumonia. The absence of the two patterns implies a poor prognosis. Finally, diffuse distribution of opacities achieved a dominant proportion in death group from the third week. Given the fact that the interval between onset of symptoms and death was short, with a median of 16 (8–33) days, the assessment of CT abnormalities should focus on the first 2 weeks.

A surprising finding was that the CT changes of our discharged patients greatly resembled those seen in SARS patients^13^. SARS-CoV-2 exhibits 79.5% sequence identity to SARS-CoV that causes SARS^20^. Both the coronavirus-associated respiratory infections are also similar in pathological features^21,22^. Xu et al.^22^ performed histological examination on a patient who died from COVID-19 on illness day 14 and this showed bilateral diffuse alveolar damage with cellular fibromyxoid exudates, which was accompanied by pulmonary edema, pneumocyte desquamation and hyaline membrane formation. Interstitial mononuclear inflammatory infiltrates, dominated by lymphocytes, were also noted in both lungs. These pathological findings indicate ARDS, and they also explain the pattern of ground-glass opacities or consolidation seen on CT within the first 2 weeks^22^. Like the findings noted in SARS patients^13^, we found that the ground-glass opacities and consolidation on the initial scans generally either resolved completely or reduced in extent or transformed into the other patterns during hospitalization. The reticular and mixed patterns were found from the second week, and they generally developed in the background of the original ground-glass opacities or consolidation and may persist for a long period. The reticular pattern might represent residual interstitial disease^12,23^, and the mixed pattern might represent mixed disease of parenchyma and interstitium during the improvement of the severe pneumonia. Precise interpretation of these CT abnormalities in COVID-19 awaits the results of further postmortem.

Like recent results^24,25^, our observations showed statistically significant increases in age, comorbidities (cerebrovascular disease, diabetes mellitus and chronic kidney disease) and clinical syndromes (sepsis and septic shock) in the dead patients compared with the discharged patients. These finding supports the belief that age, comorbidity and secondary clinical syndromes may be risk factors for poor outcomes. In terms with laboratory parameters, although the dead patients showed significant decreases in lymphocyte count, CRP, ALT and SpO_2_ level compared with the discharged patients at baseline, dynamic profile of laboratory findings merits further studies.

There are several limitations in our study. Firstly, the number and time points of CT scans were not uniform because some severely ill patients could not be weaned from mechanical ventilation, which may affect the assessment of CT changes in individuals. Secondly, residual abnormalities persisted on the last CT scan in discharged patients. Further CT follow-up is necessary to assess the long-term lung sequelae. Thirdly, the sample size of CT scans was relatively small, particularly in week 2, 3 and 4 in the dead group, which may result in an inevitable bias. Finally, all patients included were Chinese population. Based on the recent finding that the COVID-19 pathogens in Europe and the United States were more infectious due to genetic mutations^26^, the CT changes of severe COVID-19 in these countries may be different from those in China. This awaits the results of further studies.

In conclusion, this study found that the severe COVID-19 presented with characteristic CT changes and the CT changes differed between the discharged and dead patients. An understanding of these differences can be of clinical significance in the assessment of the prognosis of severely ill patients. Whether these CT changes can be used as independent predictors of prognosis awaits the results of further studies.
